# Toward personalized brain stimulation: Advances and challenges

**DOI:** 10.1111/cns.13251

**Published:** 2019-11-06

**Authors:** Hanna Lu, Linda Chiu Wa Lam, Yuping Ning

**Affiliations:** ^1^ Department of Psychiatry The Chinese University of Hong Kong Hong Kong China; ^2^ Guangdong Engineering Technology Research Center for Translational Medicine of Mental Disorders Guangzhou China; ^3^ The Affiliated Brain Hospital of Guangzhou Medical University Guangzhou China

In 1997, Freeman Dyson postulated two categories of scientific revolution in his book “Imagined Worlds”. One kind is a concept‐driven revolution, allowing us to explain “old things in new ways”; another kind is tool‐driven revolution, allowing us to discover “new things that have to be explained”.^1^ The human brain, as the most delicate and complex organ, contains around 100 billion neurons and 100 trillion connections, which are responsible for our behavior, cognition, emotion, and mind. The delicate organization of the brain can be perturbed by physical, chemical, and metabolic factors at any time point across the life span, developing brain and aging brain in particular. To explore the mystery of the brain and the possible ways for maintaining brain health and treating brain disorders, the advances of neurotechnology will be the engine of scientific revolution in 21st century.

Brain stimulation is a rapidly evolving research field which offers unique opportunities to investigate the brain function (ie, explain old things in new ways) and modulate the brain activities (ie, discover new things that have to be explained) of the targeted networks. From the perspective of bioenergetics, brain disorders are due to the disturbance of metabolism or energy. For instance, one of the most profound links between metabolism, cognition, and brain disorders is the dysfunction of mitochondria.^2^ Given the responsibility in producing the adenosine triphosphate (ATP) for energy, mitochondria use electrons and protons from molecular oxygen to reduce water and generate proton‐motive force to produce ATP from adenosine diphosphate (ADP). Since the functioning of the brain only relies on the ATP mitochondria produced, the disturbances of this process may be accompanied with brain disorders. Stemming from the dysfunction of mitochondria, recent studies found that brain stimulation could enhance the function of mitochondria and bring therapeutic benefits.^3^

**Figure 1:**
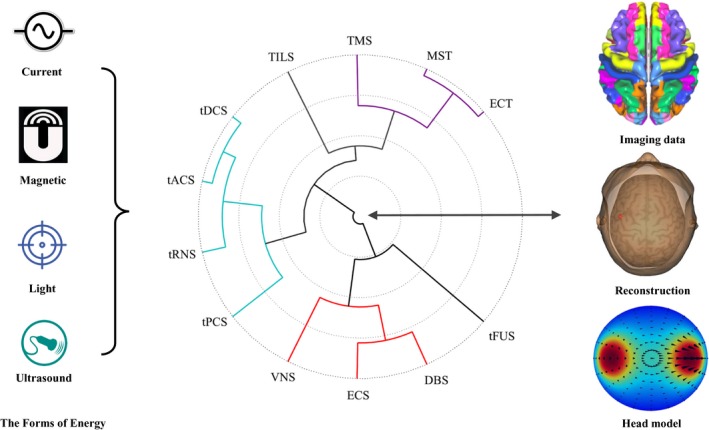
The summary of energy‐driven brain stimulation and image‐guided personalized brain stimulation. Abbreviations: TMS: Transcranial magnetic stimulation; ECT: Electroconvulsive therapy; MST: Magnetic seizure therapy; TILS: Transcranial infrared laser stimulation; DBS: Deep brain stimulation; VNS: Vagus nerve stimulation; ECS: Epidural cortical stimulation; tDCS: Transcranial direct current stimulation; tACS: Transcranial alternating current stimulation; tRNS: Transcranial random noise stimulation; tPCS: Transcranial pulsed current stimulation; and tFUS: Transcranial focused ultrasound stimulation [Colour figure can be viewed at wileyonlinelibrary.com]

From the perspective of physics (Figure 1), four types of energy, including magnetic, current, ultrasound, and light, can be transformed into the cutting‐edge stimulation technologies for delivering the energy to the targeted cortex and inducing long‐term potentiation (LTP) or long‐term depression (LTD). In the past decades, clinicians and researchers are able to use some well‐established brain stimulation techniques, such as electroconvulsive therapy (ECT), deep brain stimulation (DBS), or transcranial magnetic stimulation (TMS) for treating neurological and psychiatric disorders. Other technologies, like transcranial random noise stimulation (tRNS), transcranial direct/alternating current stimulation (tDCS/tACS), transcranial focused ultrasound stimulation (tFUS), and vagus nerve stimulation (VNS) are mainly employed as experimental tools in cognitive and clinical neuroscience, with some successful therapeutic applications in clinical trials.^4^, ^5^ In addition, novel stimulation methods, such as high‐definition transcranial direct current stimulation (HD‐tDCS), combining imaging brain stimulation, and closed‐loop brain stimulation, are the emerging and existing stimulation techniques, which need to be refined in the future studies.

For years, brain stimulation protocols are designed with the assumption that the basic molecular and cellular mechanisms that affect the targeted behavioral or cognitive changes are the same, or similar to, the biophysical processes underpinning the brain's capacity to recover from the disorders. However, beside of empirical evidence, the major challenges in the current brain stimulation studies should be noted as well: (a) Heterogeneity: due to the increased inter‐individual and intra‐individual variability in developing, aging, and clinical populations, causal inference with observational data is nearly impossible without strong assumptions; (b) Consideration of key parameters: scalp‐to‐cortex distance (SCD), as one of the technological and fundamental parameters of neuroimaging and noninvasive brain stimulation, has been highlighted in the updated guidelines.^6^ Recent studies have found significant increased SCD in senior adults and dementia patients 7; (c) Sham stimulation: considering the placebo effects of brain stimulation, sham/control condition is designed to mimic the skin sensations of active stimulation, such as tingling or itching, somewhat easier in tDCS than in TMS, but may not be sufficient. Richardson and colleagues compared several protocols of sham stimulation and found that none of the sham conditions were entirely equivalent to the verum stimulation in sensation rating 8; and (d) Safety: any technique that involves delivering magnetic or electrical stimulation to the brain must consider safety. Several reports have mentioned the skin burns (tDCS), pain, and headache (TMS and ECT) during the treatment of brain stimulation.^9^, ^10^ However, standardized protocol to monitor and record the impedance and potential side effects is still lacking.

Taken together, this special issue on Toward Personalized Brain Stimulation: Advances and Challenges aims to highlight the recent progress of brain stimulation in clinical practice and further deepens our understanding the mechanisms of stimulation‐induced function changes underlying cognitive dysfunction in neurological and psychiatric disorders, and therefore investigating and developing personalized brain stimulation interventions. In this special issue, we have assembled reviews and original articles by the researchers and clinicians who have lead the way in the treatment of brain disorders and in the development of computational head model for the personalized brain stimulation. Finally, the guest editors would like to thank CNSNT and its editor in chief, *Ding‐Feng Su* and managing editor, *Buddy Zhou* for providing initial impetus for compiling this special issue. We also thank all the contributors who share their insightful thoughts and recent research findings.
